# A machine learning study of the effect of thrombolysis on outcome at discharge in the UK stroke registry: how does benefit compare with clinical trials?

**DOI:** 10.1093/esj/aakag078

**Published:** 2026-07-13

**Authors:** Kerry Pearn, Michael Allen, Anna Laws, Peter McMeekin, Richard Everson, Mark Kelson, Jason Scott, Eleanor Kashouris, Joao Delgado, Leon Farmer, Lauren Asare, Ajay Bhalla, Martin James

**Affiliations:** University of Exeter Medical School, Exeter, United Kingdom; NIHR South West Peninsula Applied Research Collaboration (ARC), Exeter, United Kingdom; University of Exeter Medical School, Exeter, United Kingdom; NIHR South West Peninsula Applied Research Collaboration (ARC), Exeter, United Kingdom; University of Exeter Medical School, Exeter, United Kingdom; NIHR South West Peninsula Applied Research Collaboration (ARC), Exeter, United Kingdom; University of Northumbria, Newcastle, United Kingdom; University of Exeter, Exeter, United Kingdom; University of Exeter, Exeter, United Kingdom; University of Northumbria, Newcastle, United Kingdom; University of Northumbria, Newcastle, United Kingdom; University of Exeter Medical School, Exeter, United Kingdom; University of Exeter Medical School, Exeter, United Kingdom; NIHR South West Peninsula Applied Research Collaboration (ARC), Exeter, United Kingdom; University of Exeter Medical School, Exeter, United Kingdom; NIHR South West Peninsula Applied Research Collaboration (ARC), Exeter, United Kingdom; Guy’s and St Thomas’ NHS Foundation Trust, London, United Kingdom; University of Exeter Medical School, Exeter, United Kingdom; NIHR South West Peninsula Applied Research Collaboration (ARC), Exeter, United Kingdom

**Keywords:** stroke, alteplase, ischemic stroke, health service research, machine learning, thrombolysis

## Abstract

**Introduction:**

Thrombolysis has been shown to reduce disability after acute ischaemic stroke in randomised trials. Our study sought to compare the benefit from thrombolysis observed in a comprehensive national stroke registry with that expected from the clinical trials.

**Patients and methods:**

Data from a total of 168,347 ischaemic stroke patients who attended one of 118 emergency stroke hospitals in England and Wales from 2016 to 2021 were extracted from the Sentinel Stroke National Audit Programme. We constructed a machine learning model, designed to isolate the effect of thrombolysis, using explainable machine learning (XGBoost with SHapley Additive exPlanations [SHAP]).

**Results:**

Thrombolysis was found to be associated with a statistically significant improvement in the odds of achieving a better outcome (modified Rankin scale [mRS] threshold). Regression analysis predicted a maximum 2.5-fold improvement in odds of achieving mRS 0–1, with a decline to no treatment effect at 5 h 28 min post-onset.

**Discussion:**

Our results confirm a beneficial effect of thrombolysis in a large prospective national stroke registry, and align closely with meta-analyses of clinical trials of thrombolysis both in terms of magnitude of effect and decline over time. This work also demonstrates the potential to apply explainable machine learning to observational data to assist in understanding how clinical trials are implemented in real-world settings.

**Conclusions:**

Thrombolysis, in practice, has the same observed benefit as in the clinical trials.

## Graphical Abstract

**Graphical Abstract:**
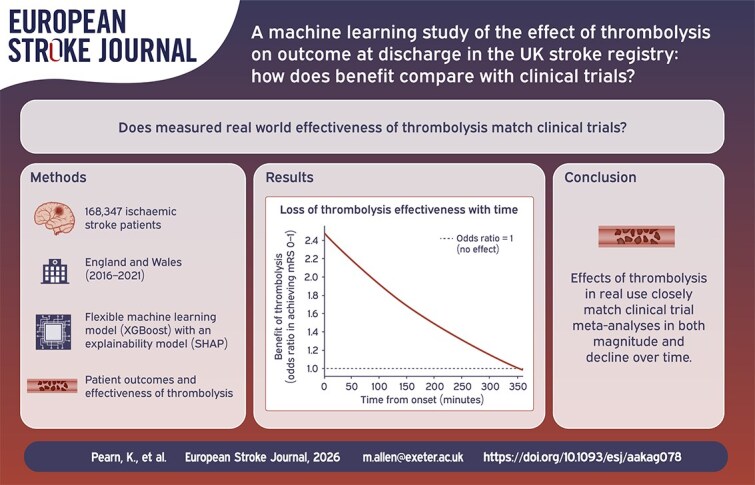

## Introduction

Stroke remains one of the top three global causes of death and disability.^1^ Despite reductions in age-standardised rates of stroke, ageing populations are driving an increase in the absolute number of strokes.^1^

Thrombolysis with recombinant tissue plasminogen activator, can significantly reduce disability after ischaemic stroke, provided it is given in the first few hours after stroke onset.^2^ Despite thrombolysis being of proven benefit in ischaemic stroke, use of thrombolysis varies significantly both between and within European countries.^3^ In England and Wales the prospective Sentinel Stroke National Audit Programme (SSNAP; the national stroke registry) reported that in 2021/22, 20 years after the original European Medicines Agency licencing of alteplase for acute ischaemic stroke, thrombolysis rates varied from 1% to 28% of emergency stroke admissions between hospitals,^4^ with a median rate of 10.4% and an interquartile range of 8%–13%, against a 2019 National Health Service (NHS) England long-term plan that 20% of patients with stroke should be receiving thrombolysis.^5^

Studies have shown that the reasons for low and varying thrombolysis use are multifactorial. Organisational factors can reduce the effectiveness of thrombolysis administration,^3^ and clinicians can have varying attitudes to use of thrombolysis.^6^

We have previously used clinical pathway simulation and machine learning modelling of the variation in thrombolysis use between stroke teams to establish in England and Wales. We found that the largest contributor to variation in thrombolysis practice was the attitudes of clinicians to the use of thrombolysis, particularly in “less than ideal” patients.^7^^,^^8^

In addressing such persisting uncertainty among clinicians, it is important to test that the benefit of thrombolysis predicted in clinical trials is being observed in the context of real-world stroke care. Various observational studies have provided further support for the effectiveness of thrombolysis.^9^^,^^10^ None of these studies, however, was able to investigate the relationship between time-to-thrombolysis and effectiveness compared with the clinical trial meta-analyses, and none was in a specific context where clinical registry data are used to drive change.

With the prospective collection of clinical data into SSNAP for all emergency stroke admissions in all hospitals in England and Wales, and with the power of new explainable machine learning techniques, our aim was to build a model of stroke outcomes, perform a large scale analysis of the benefit of thrombolysis in practice in a UK setting, and to compare the relationship between time-to-thrombolysis and effectiveness with clinical trial results.

## Methods

### Data

Data were retrieved for 168,347 emergency ischaemic stroke admissions to hospitals in England and Wales for six calendar years, 2016–2021 inclusive, obtained from the national stroke registry SSNAP. Further details can be found in the Supplementary material.

### Machine learning models

We trained a set of six independent XGBoost models^11^ to predict the likelihood of an ischaemic stroke patient attaining any given modified Rankin scale (mRS) threshold at discharge (where achieving a lower threshold is better). The model of mRS *≤*5 also provides a model of survival vs. death. The results from the test set for the model fitted on the first k-fold split was used to report the relationships between feature values and their contribution to the predictions.

Code, and full results, for the machine learning work can be found at: https://github.com/samuel-book/thrombolysis_clinical_trials_ml_paper. Further details of method development can be found in the Supplementary material.

### Feature selection and model building

The complete available data set contained 57 features that described patient demographic and clinical characteristics, the acute stroke pathway, the use/time of thrombolysis and the stroke team attended. We have previously identified features that influence the choice to use thrombolysis.^12^ For the current work, we identified the features that influence patient outcome and, after discussions with clinicians, combined the two sets of features (on use of thrombolysis and outcome after stroke) into a proposed model with seven features that would better isolate the effect of thrombolysis on outcome.

### Model accuracy

For each of the six machine learning models, we report the model accuracy measured using 5-fold cross-validation where the data was split into five different 80:20 train/test splits with each patient being in one, and only one, test set.

### SHapley Additive exPlanation values

SHapley Additive exPlanation (SHAP) values quantify the contribution of each feature to the model’s prediction of a good discharge outcome.^13^ Because SHAP values are expressed on the log-odds scale, they are additive and therefore more suitable than probabilities for assessing the contribution of individual features to the final prediction. Separate SHAP analyses were performed for each of the six machine learning models, corresponding to the six mRS thresholds. A positive SHAP value indicates that a feature increased the likelihood of reaching the mRS threshold (better outcome), whereas a negative SHAP value indicates that a feature decreased it. SHAP values can be examined both locally, at the individual patient level, and globally, across the cohort, to identify general patterns in how patient, pathway, and hospital characteristics were associated with discharge outcomes.

### Counterfactual treatment: what if patients had not received thrombolysis?

After constructing a model (splitting data 80% training, 20% test) to isolate the effect of thrombolysis from confounding variables, we used counterfactuals for each patient in the test set who had received thrombolysis, to assess the contribution that thrombolysis made on the likelihood of achieving any particular mRS threshold at discharge. The counterfactual result was achieved by running the same model, but changing the patient to mimic not receiving thrombolysis.

The predicted contribution of thrombolysis to the achievement of an excellent outcome (mRS 0–1) was estimated by the difference in SHAP thrombolysis values between when thrombolysis was administered and the counterfactual scenario of not receiving thrombolysis. To model decay of thrombolysis benefit with treatment delay, we fitted a linear regression of estimated thrombolysis benefit (log-odds shift for achieving mRS 0–1) on time from onset to thrombolysis, excluding patients treated after 300 min. We defined an excellent outcome as mRS 0–1 to align with the definition used in the clinical trial meta-analysis by Emberson et al.^2^ Analyses were performed in three thrombolysed cohorts: (1) all ischaemic strokes (*n* = 6897), (2) severe ischaemic strokes (National Institutes of Health Stroke Scale [NIHSS] *≥*11; *n* = 2897) and (3) mild-to-moderate ischaemic strokes (NIHSS 0–10; *n* = 4000).

## Results

### Descriptive statistics

A total of 168,347 patients (with onset-to-scan of within 255 min) attended 118 hospitals with total stroke admissions per hospital ranging from 358 to 4559, and with hospital thrombolysis rates ranging from 5.9% to 39.2%. Of the 133,516 patients who did not receive thrombolysis, their range of disability at discharge was: 12% mRS 0, 33% mRS 0–1, 52% mRS 0–2, 69% mRS 0–3, 82% mRS 0–4 and 88% mRS 0–5 at discharge. Of the 34,831 patients who received thrombolysis, their range of disability at discharge was: 15% mRS 0, 35% mRS 0–1, 54% mRS 0–2, 69% mRS 0–3, 81% mRS 0–4 and 86% mRS 0–5. Table 1 shows further descriptive statistics for patients who received or did not receive thrombolysis. A total of 30.7% had 6-month follow-up assessment of mRS. On average, mRS at 6 months was very close to mRS at discharge (0.12 higher), but there was variation at individual patient level: 31% had no change between discharge and 6-month follow-up, 73% had no change or a change of 1 mRS band, and 27% had a change of two or more mRS bands. Those with discharge mRS of 2–4 were more likely to improve, rather than worsen. Further descriptive statistics are provided in the Supplementary material.

**Table 1:** Descriptive statistics for patients receiving or not receiving thrombolysis.

	***n***	**Thrombolysis given**		***n***	**Thrombolysis not given**	
		**Mean (SD)**	**Median (IQR)**		**Mean (SD)**	**Median (IQR)**
**Age, years**	41,163	72.3 (13.6)	72.5 (62.5–82.5)	315,941	74.5 (13.3)	77.5 (67.5–82.5)
**Infarction**	41,163	1 (NA)	NA	315,941	0.86 (NA)	NA
**Stroke severity**	41,163	11.0 (7.1)	9 (5–16)	315,941	6.6 (7.6)	4 (2–8)
**Prior disability**	41,163	0.71 (1.17)	0 (0–1)	315,941	1.08 (1.39)	0 (0–2)
**Male**	41,163	0.56 (NA)	NA	315,941	0.53 (NA)	NA
**Onset known**	41,163	0.99 (NA)	NA	315,941	0.63 (NA)	NA
**Precise onset known**	41,163	0.80 (NA)	NA	315,941	0.28 (NA)	NA
**Onset during sleep**	41,163	0.01 (NA)	NA	315,941	0.15 (NA)	NA
**Onset to arrival time**	40,583	102 (53)	90 (66–128)	198,105	57 (1115)	250 (112–713)
**Arrive by ambulance**	41,161	0.92 (NA)	NA	315,934	0.77 (NA)	NA
**Arrival to scan time**	41,163	23 (20)	19 (11–29)	315,941	217 (2016)	61 (26–149)
**Congestive heart failure**	41,163	0.04 (NA)	NA	315,941	0.05 (NA)	NA
**Hypertension**	41,163	0.51 (NA)	NA	315,941	0.55 (NA)	NA
**Afib**	41,163	0.12 (NA)	NA	315,941	0.19 (NA)	NA
**New Afib diagnosis**	24,485	0.11 (NA)	NA	172,727	0.06 (NA)	NA
**Diabetes**	41,163	0.17 (NA)	NA	315,941	0.22 (NA)	NA
**Prior stroke or TIA**	41,163	0.20 (NA)	NA	315,941	0.26 (NA)	NA
**Afib antiplatelet**	41,163	0.03 (NA)	NA	315,940	0.03 (NA)	NA
**Afib anticoagulant**	30,128	0.04 (NA)	NA	237,923	0.19 (NA)	NA
**Afib vitamin K anticoagulant**	41,163	0.01 (NA)	NA	315,940	0.03 (NA)	NA
**Afib DOAC anticoagulant**	41,163	0.01 (NA)	NA	315,940	0.08 (NA)	NA
**Scan to thrombolysis time**	41,163	37 (29)	31 (19–47)	NA	NA	NA
**Thrombectomy**	41,163	0.06 (NA)	NA	315,941	0.01 (NA)	NA
**Arrival to thrombectomy time**	2591	201 (179)	171 (114–233)	1752	249 (278)	172 (104–269)

Afib, atrial fibrillation; DOAC, direct oral anticoagulants; IQR, interquartile range; TIA, transient ischaemic attack. NA, not applicable for a proportion.

### Feature selection and proposed model

**Figure 1:**
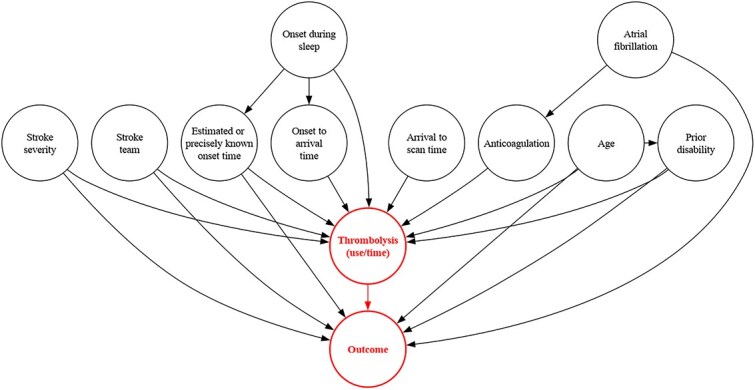
A directed acyclic graph (DAG) showing the proposed relationships in propensity to use thrombolysis and outcome after stroke with or without thrombolysis.

Features affecting choice of thrombolysis have previously been described.^12^ Details of feature selection for outcome prediction are detailed in the Supplementary material. Based also on the selection of characteristics for the prediction of the outcome, we developed Figure 1 as a as a proposed schematic of the factors that affect the use of thrombolysis and the factors that directly affect the outcome. This proposed causal model is based on features identified as affecting outcome or propensity to use thrombolysis, correlations present in the data, known temporal relationships in the data and clinical review.

In order to isolate the effect of thrombolysis appropriately, we predicted outcome based on use/time to thrombolysis, and six factors identified as confounding variables (affecting both outcome directly and propensity to use thrombolysis):

1. Onset to thrombolysis time
2. Prior disability level: mRS before stroke
3. Stroke severity: NIHSS on arrival
4. Stroke team: hospital first attended
5. Age: as midpoint of 5-year age bands
6. Atrial fibrillation diagnosis: patient had a diagnosis of atrial fibrillation either on arrival or diagnosed during admission
7. Precise onset known: onset time recorded was recorded as being precise time (as opposed to a best estimate)

### Model accuracy

With these seven input features, the overall accuracy ranged from 77.6% to 89.7% in the six mRS disability level models (the SD of accuracy across the five k-fold train/test splits ranged from 0.1% to 0.2%). This represented between 95% and 98% of the accuracy that is obtained with all the features as inputs. Receiver operating characteristic area under the curve (ROC-AUC) ranged from 0.852 to 0.893 across the six models (SD of the ROC-AUC across the five k-folds ranged from 0.001 to 0.002). Results were reproducible across the five k-folds, so subsequent analysis was performed on the first k-fold split.

### SHAP patterns

**Figure 2:**
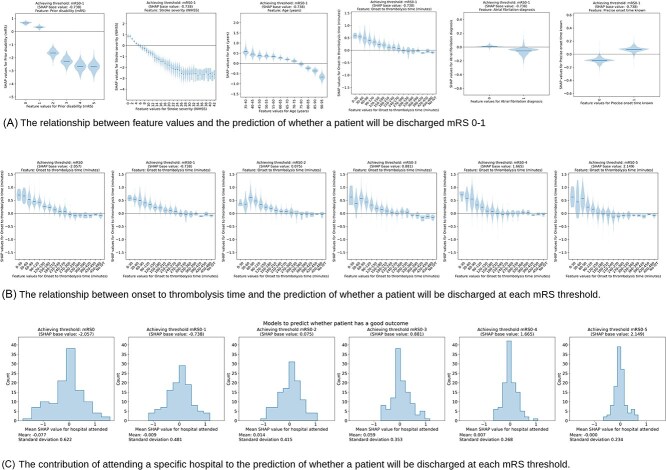
The relationship between feature values and SHapley Additive exPlanations (SHAP) values in predicting whether a patient is discharged with mRS 0-1 (panel A), or at each modified Rankin scale (mRS) threshold (panels B and C). Violin plots show the distribution of SHAP values across the patient population for each feature value, with the mid-line showing the median SHAP value.

The SHAP values were calculated for each feature for each patient, showing how each value affects the log odds of achieving any particular mRS threshold at discharge. For each patient in the test set we extracted SHAP values for each feature with its corresponding feature value. Figure 2A shows the relationship between feature values and the odds of a patient having an excellent outcome (mRS 0–1) at discharge (a positive SHAP value contributes to an increased likelihood, and a negative SHAP value contributes to a reduced likelihood). We found that low prior disability, lower stroke severity, younger age, receiving thrombolysis (and receiving it sooner after onset) and having no diagnosis of atrial fibrillation contributed to a patient more likely having an excellent outcome at discharge. Figure 2B focusses on the feature onset to thrombolysis time, and its effect on reaching any disability threshold. As time from onset to thrombolysis increased, the beneficial effect of thrombolysis decayed. For thresholds up to, and including, mRS 3, the median effect of thrombolysis remained positive, compared with not receiving thrombolysis, up to the maximum time of 720 min. For thresholds of mRS 4 and upwards, the median effect of thrombolysis became negative at longer onset-to-thrombolysis times. This was especially apparent in how thrombolysis changed the overall odds of survival (mRS 0–5) where the median effect of thrombolysis became negative from about 155 min. Later thrombolysis may therefore increase the proportion of patients attaining mRS 0–3, but at the cost of some reduction in overall survival. Earlier thrombolysis improved the odds of survival. Figure 2C shows the contribution from attending a specific hospital to predicting whether the patient achieved each mRS threshold at discharge. As the mRS threshold becomes more inclusive of higher disability levels, the effect of the hospital attended had a reduced contribution. In other words, the hospital attended made a greater contribution to variation in outcome with thrombolysis for patients with lesser degrees of disability after treatment.

### Counterfactuals: what if patients had not received thrombolysis?

**Figure 3:**
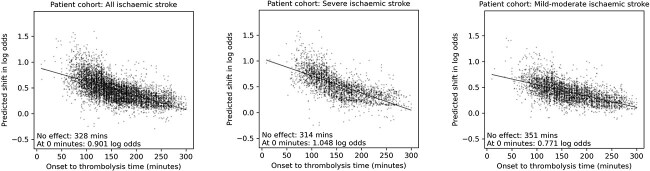
Linear regression of thrombolysis contribution to excellent outcome (modified Rankin scale [mRS] 0–1) at discharge as a function of onset-to-thrombolysis time (OTT) in patients treated within 300 min. Left: all treated stroke patients (*n* = 6796); middle: severe stroke patients (National Institutes of Health Stroke Scale [NIHSS] 11+; *n* = 2856); right: mild–moderate stroke patients (NIHSS 0–10; *n* = 3940).

Using the counterfactual results for all of the patients in the first k-fold test set that received thrombolysis within 300 min from stroke onset (*n* = 6796), Figure 3 shows a plot of the contribution of time-to-thrombolysis towards being discharged with mRS 0-1. Table 2 shows a linear regression fitted to the shift in the contribution of thrombolysis towards having an excellent outcome at discharge (mRS 0–1) with respect to the onset to thrombolysis time. We found, for all treated patients, that the effect of thrombolysis had declined to zero at 328 min, and the effect from thrombolysis was improving log odds of being discharged mRS 0–1 by 0.90 if it were, theoretically, given at the time of stroke onset. We observed that the maximum theoretical effect of thrombolysis (if given at time of stroke onset) was greater for the severe stroke group (1.048 log odds) than the mild–moderate stroke group (0.771 log odds). However, the effect of thrombolysis declined more rapidly in the severe stroke group, reaching no effect at 314 min for severe stroke patients, compared with 351 min for mild–moderate stroke patients.

**Table 2:** Linear regression of the relationship between OTT and the improvement in log odds of achieving an excellent outcome (mRS 0–1), stratified by stroke severity.

**Ischaemic stroke type**	**Parameter**	**Estimate**	**SE**	**Low 95% confidence**	**High 95% confidence**	***t***	***P***
**All**	Constant	0.901	0.007	0.888	0.915	132.5	.000
	OTT (min) coef	−0.0027	4.04e-05	−0.003	−0.003	−68.1	.000
**Severe (NIHSS 11+)**	Constant	1.048	0.011	1.026	1.069	96.7	.000
	OTT (min) coef	−0.0033	6.67e-05	−0.003	−0.003	−50.0	.000
**Mild–moderate (NIHSS 0–10)**	Constant	0.771	0.008	0.755	0.786	97.6	.000
	OTT (min) coef	−0.0022	4.57e-05	−0.002	−0.002	−48.1	.000

NIHSS, National Institutes of Health Stroke Scale; OTT, onset-to-thrombolysis time;

## Discussion

In this study we have built explainable machine learning models based on a comprehensive prospective national stroke registry capable of predicting outcome, at any given disability threshold, with high precision. The models were constructed considering features which affect both the use and outcome of thrombolysis, in order to isolate the effect of thrombolysis and to understand the effect of thrombolysis in the real world.

The most influential factors that favoured achieving any given disability threshold were lower pre-stroke disability, lower stroke severity, younger age, and the use and speed of thrombolysis. The hospital attended also had an effect on achieving any disability threshold, although that effect was reduced among the more severely affected patients with higher disability at discharge. This could be due to (1) some hospitals discharging patients earlier with more disability, eg, those with more community rehabilitation available, (2) effects of other hospital treatments on outcomes, eg, better/worse stroke unit care, or (3) hospitals assessing disability at discharge differently. From our model we cannot speculate further, but by including stroke team in the model we can adjust the model for these effects, thereby allowing a clearer view of other patient or organisational features, including use and time of thrombolysis, affecting outcome.

Using a large, inclusive real-world model enables counterfactual prediction of outcomes with and without thrombolysis for any patient. Here, we focused on predicting outcomes without thrombolysis among patients who actually received it, allowing estimation of the treatment effect in treated patients. SHAP values were used to isolate the contribution of thrombolysis to the model prediction. SHAP values provide the contribution of any given input to the model to the models’ final prediction (eg, of the patient being discharged mRS 0–1), and the model allowed us to isolate the effect of thrombolysis from other patient features, and run counterfactual analysis of what the outcome would have been if a patient had not received thrombolysis (when they had received it). We found thrombolysis improved the odds of being at or below any given disability threshold, but the effect of thrombolysis was reduced, and decayed to no-effect sooner, for outcomes of mRS ≤5 (survival). We found that the effect of thrombolysis was present for both mild–moderate and severe strokes, but with a larger effect in severe strokes. The findings from this study were remarkably similar to the meta-analysis of clinical trials,^2^ which extrapolate back to a 0.69 log odds improvement (equivalent to an odds ratio of 2.0) of being mRS 0–1 at stroke onset, with no effect after 378 min (6.3 h). Our maximum theoretical improvement in log odds of achieving mRS 0–1 was a little higher at 0.90 (equivalent to an odds ratio of 2.5), with a decline to no-effect at 328 min (5.5 h), indicating a slightly faster decline in effectiveness with time in our observational real-world study when compared with the original randomised data. This is possibly due to the licencing conditions for alteplase, in that the meta-analysis included randomised trials out to 6 h from stroke onset,^2^ whereas the overwhelming majority of patients treated in the UK over the 6-year study period were restricted to guideline-based eligibility within 4.5 h.^14^

The clinical significance of this work is that we have confirmed the net benefit of thrombolysis in a very large prospective national stroke registry that encompasses patients outside the strictly defined eligibility of the original randomised trials, and used sophisticated machine learning with SHAP to isolate the effect of thrombolysis from other patient characteristics that influence outcomes. This provides significant reassurance that in real-world clinical practice, the anticipated benefits of thrombolysis are being delivered in the context of the stroke system in England and Wales, even when treatment is implemented in a wider spectrum of patients than those represented in the randomised trials.

In summary, applying explainable machine learning to an observational data set demonstrated that the effectiveness of thrombolysis in the real world appears to be at least as good as the clinical trials indicated. Our results should give stroke clinicians more confidence that the beneficial effect of thrombolysis is seen in real treatment populations. The size of the observational data set allows for more detailed analysis of the benefit of thrombolysis in subgroups of patients.

### Study limitations

Alhough we are using a very large data set, our study necessarily has some limitations:

- Our results are specific to real-world use of thrombolysis in hospitals in England and Wales. They are not intended to be generalisable outside of this area.
- A group of more severely affected stroke patients who proceeded to receive thrombectomy has been excluded from the analysis, although during the 6-year study period endovascular intervention was increasing from a low base in the UK, and increased from 0.7% to 2.0% of all stroke.
- We have used the mRS at hospital discharge as our primary outcome, whereas the trials used the mRS at 90 days. On average across the patient population stroke outcomes are similar at 3 months^15^ and at 6 months (our own analysis) but individual patients may improve or worsen, though those discharged with mRS 2–4 are more likely to improve than to worsen. Although we do not include discharge timing directly in the model, by using stroke team identifiers, the model should adjust for differences that are due to between-hospital variation in discharge practices, such as timing of discharge.
- Our results are based on data from 2016 to 2021. Since then, practices may have changed, such as more use of tenecteplase rather than alteplase, more use of thrombectomy and increased use of anti-platelet therapy. When interpreting results, this study should be seen in the context of largely studying alteplase. Further studies, with more recent data, should be performed for an analysis of more recent treatment regimes.
- The directed acyclic graph (DAG) was constructed internally within the team without external validation and was based on routine (observed) data meaning that we did not include unobserved variables. Though we have sought the best isolation of the effect of thrombolysis that we can, it is possible that unknown or unmeasured confounders may be skewing results.

### Further work

Considering recent suggestions that thrombolysis should not be used in mild stroke,^16^^,^^17^ a more in-depth study of the observed effect of thrombolysis mild stroke, combining machine learning with causal inference methods such as propensity-score adjustment or target trial emulation, would add to the evidence base around in this contentious group of patients. Additionally, the methods described here could be applied to looking at level of benefit in other subgroups of patients (eg, where prior disability exists). Qualitative research with clinicians involved in thrombolysis decisions would provide an opportunity to examine our causal assumptions. Elicitation of clinician views would also provide an opportunity to examine whether any unobserved variables should be included to provide a more comprehensive DAG.

## Supplementary Material

supplement_aakag078
