# Supplementary material for: A machine learning study of the effect of thrombolysis on outcome at discharge in the UK stroke registry: how does benefit compare with clinical trials?
Source: Eur Stroke J. 2026 Jul 13;11(7):aakag078. doi: 10.1093/esj/aakag078 (PMC13358869; doi:10.1093/esj/aakag078)
Supplement: supplement_aakag078 [file supplement_aakag078.pdf]

Supplementary material: A causal machine learning study of the effect of thrombolysis on outcome at discharge in the UK stroke registry: How does benefit compare with clinical trials?

S1 Data

Data were retrieved for 168,347 emergency ischaemic stroke admissions, arriving by ambulance, at acute stroke teams in England for six years, 2016–2021 (inclusive), obtained from the Sentinel Stroke National Audit Programme (SSNAP). The registry prospectively collects clinical data from 100% of acute hospitals in England and Wales, with case ascertainment estimated at >90% when compared with administrative coding data. Data fields were provided for the hyper-acute phase of the stroke pathway, up to and including our target feature: disability on inpatient discharge. Disability is recorded in the SSNAP dataset using the modified Rankin Scale (mRS), where mRS 0 represents perfect health and mRS 6 represents death. The data includes 118 acute stroke hospitals (each has at least 250 stroke admissions and delivers thrombolysis to at least 10 patients in the six year study period). Patients who went on to receive thrombectomy were excluded from the study (rates of thrombectomy recorded in SSNAP increased from 0.7% to 2.0% of all stroke during this period). Data preparation prior to sharing by the national stroke registry restricts the description of age to within 5-year age bands.

S1.1 Data completion

Table S2 shows data completion for all original data, and table S3 shows data used for machine learning study.

Table S1: Data completeness for all data

Variable	Total Patients	Missing Values	Missing (%)	Complete (%)
PatientId	360,381	0	0.000	100.000
ProClinV1Id	360,381	0	0.000	100.000
TeamName	360,381	0	0.000	100.000
AgeUnder40	360,381	0	0.000	100.000
Age40to44	360,381	0	0.000	100.000
Age45to49	360,381	0	0.000	100.000
Age50to54	360,381	0	0.000	100.000
Age55to59	360,381	0	0.000	100.000
Age60to64	360,381	0	0.000	100.000
Age65to69	360,381	0	0.000	100.000
Age70to74	360,381	0	0.000	100.000
Age75to79	360,381	0	0.000	100.000
Age80to84	360,381	0	0.000	100.000
Age85to89	360,381	0	0.000	100.000
AgeOver90	360,381	0	0.000	100.000
S1Gender	360,381	0	0.000	100.000
OnsettoArrivalMinutes	360,381	0	0.000	100.000
S1OnsetDateType	360,381	0	0.000	100.000
S1OnsetTimeType	360,381	0	0.000	100.000
S1ArriveByAmbulance	360,381	0	0.000	100.000
FirstArrivalMonthYear	360,381	0	0.000	100.000
FirstArrivalWeekday	360,381	0	0.000	100.000

Continued on next page

Table S1 – *continued from previous page*

Variable	Total Patients	Missing Values	Missing (%)	Complete (%)
FirstArrivalTime	360,381	0	0.000	100.000
S2CoMCongestiveHeartFailure	360,381	0	0.000	100.000
S2CoMHypertension	360,381	0	0.000	100.000
S2CoMAtrialFibrillation	360,381	0	0.000	100.000
S2CoMDiabetes	360,381	0	0.000	100.000
S2CoMStrokeTIA	360,381	0	0.000	100.000
S2CoMAFAntiplatelet	360,381	294,785	81.798	18.202
S2CoMAFAnticoagulent	360,381	90,047	24.987	75.013
S2CoMAFAnticoagulentVitK	360,381	112,376	31.183	68.817
S2CoMAFAnticoagulentDOAC	360,381	112,376	31.183	68.817
S2CoMAFAnticoagulentHeparin	360,381	112,376	31.183	68.817
S2INR	360,381	351,875	97.640	2.360
S2INRHigh	360,381	112,376	31.183	68.817
S2INRNK	360,381	112,376	31.183	68.817
S2NewAFDiagnosis	360,381	161,406	44.788	55.212
S2RankinBeforeStroke	360,381	0	0.000	100.000
S2NihssArrival	360,381	0	0.000	100.000
S2NihssArrivalLoc	360,381	0	0.000	100.000
S2NihssArrivalLocQuestions	360,381	0	0.000	100.000
S2NihssArrivalLocCommands	360,381	0	0.000	100.000
S2NihssArrivalBestGaze	360,381	0	0.000	100.000
S2NihssArrivalVisual	360,381	0	0.000	100.000
S2NihssArrivalFacialPalsy	360,381	0	0.000	100.000
S2NihssArrivalMotorArmLeft	360,381	0	0.000	100.000
S2NihssArrivalMotorArmRight	360,381	0	0.000	100.000
S2NihssArrivalMotorLegLeft	360,381	0	0.000	100.000
S2NihssArrivalMotorLegRight	360,381	0	0.000	100.000
S2NihssArrivalLimbAtaxia	360,381	0	0.000	100.000
S2NihssArrivalSensory	360,381	0	0.000	100.000
S2NihssArrivalBestLanguage	360,381	0	0.000	100.000
S2NihssArrivalDysarthria	360,381	0	0.000	100.000
S2NihssArrivalExtinctionInattention	360,381	0	0.000	100.000
ArrivaltoBrainImagingMinutes	360,381	1,388	0.385	99.615
S2StrokeType	360,381	1,388	0.385	99.615
S2Thrombolysis	360,381	0	0.000	100.000
S2ThrombolysisNoReason	360,381	358,912	99.592	0.408
S2ThrombolysisNoButHaemorrhagic	360,381	0	0.000	100.000
S2ThrombolysisNoButTimeWindow	360,381	0	0.000	100.000
S2ThrombolysisNoButComorbidity	360,381	0	0.000	100.000
S2ThrombolysisNoButMedication	360,381	0	0.000	100.000
S2ThrombolysisNoButRefusal	360,381	0	0.000	100.000
S2ThrombolysisNoButAge	360,381	0	0.000	100.000
S2ThrombolysisNoButImproving	360,381	0	0.000	100.000
S2ThrombolysisNoButTooMildSevere	360,381	0	0.000	100.000
S2ThrombolysisNoButTimeUnknownWakeUp	360,381	0	0.000	100.000

Continued on next page

Table S1 – *continued from previous page*

Variable	Total Patients	Missing Values	Missing (%)	Complete (%)
S2ThrombolysisNoButOtherMedical	360,381	0	0.000	100.000
ArrivaltoThrombolysisMinutes	360,381	319,218	88.578	11.422
S2TIAInLastMonth	360,381	332,601	92.291	7.709
ArrivaltoArterialPunctureMinutes	360,381	356,038	98.795	1.205
S7DischargeType	360,381	2,469	0.685	99.315
ArrivalToDeathDays	360,381	310,324	86.110	13.890
S7StrokeUnitDeath	360,381	314,809	87.354	12.646
S7RankinDischarge	360,381	2,576	0.715	99.285
S8Rankin6Month	360,381	250,658	69.554	30.446
S8Rankin6MonthNK	360,381	133,399	37.016	62.984
CallConnectedtoArrivalMinutes	360,381	251,896	69.897	30.103
ArrivalPatientLocationtoArrivalMinutes	360,381	251,900	69.898	30.102
DeparturePatientLocationtoArrivalMinutes	360,381	251,991	69.923	30.077
WheelsStoptoArrivalMinutes	360,381	267,405	74.201	25.799
MobileDatatoArrivalMinutes	360,381	344,310	95.541	4.459
S1PreHospitalImpression	360,381	252,031	69.935	30.065

Table S2: Data completeness for restricted machine learning data set

Variable	Total Patients	Missing Values	Missing (%)	Complete (%)
id	168,347	0	0.000	100.000
stroke_team	168,347	0	0.000	100.000
age	168,347	0	0.000	100.000
male	168,347	0	0.000	100.000
onset_to_arrival_time	168,347	0	0.000	100.000
precise_onset_known	168,347	0	0.000	100.000
onset_during_sleep	168,347	0	0.000	100.000
month	168,347	0	0.000	100.000
year	168,347	0	0.000	100.000
weekday	168,347	0	0.000	100.000
arrival_time_3_hour_period	168,347	0	0.000	100.000
arrival_to_scan_time	168,347	0	0.000	100.000
scan_to_thrombolysis_time	168,347	0	0.000	100.000
congestive_heart_failure	168,347	0	0.000	100.000
hypertension	168,347	0	0.000	100.000
atrial_fibrillation	168,347	0	0.000	100.000
diabetes	168,347	0	0.000	100.000
prior_stroke_tia	168,347	0	0.000	100.000
afib_antiplatelet	168,347	0	0.000	100.000
afib_anticoagulant	168,347	0	0.000	100.000
new_afib_diagnosis	168,347	0	0.000	100.000
any_afib_diagnosis	168,347	0	0.000	100.000
prior_disability	168,347	0	0.000	100.000

Continued on next page

Table S2 – *continued from previous page*

Variable	Total Patients	Missing Values	Missing (%)	Complete (%)
stroke_severity	168,347	0	0.000	100.000
nihss_complete	168,347	0	0.000	100.000
nihss_arrival_loc	168,347	0	0.000	100.000
nihss_arrival_loc_questions	168,347	2,802	1.664	98.336
nihss_arrival_loc_commands	168,347	2,610	1.550	98.450
nihss_arrival_best_gaze	168,347	3,258	1.935	98.065
nihss_arrival_visual	168,347	4,264	2.533	97.467
nihss_arrival_facial_palsy	168,347	2,645	1.571	98.429
nihss_arrival_motor_arm_left	168,347	2,612	1.552	98.448
nihss_arrival_motor_arm_right	168,347	2,615	1.553	98.447
nihss_arrival_motor_leg_left	168,347	2,748	1.632	98.368
nihss_arrival_motor_leg_right	168,347	2,769	1.645	98.355
nihss_arrival_limb_ataxia	168,347	4,723	2.806	97.194
nihss_arrival_sensory	168,347	4,119	2.447	97.553
nihss_arrival_best_language	168,347	2,775	1.648	98.352
nihss_arrival_dysarthria	168,347	3,295	1.957	98.043
nihss_arrival_extinction_inattention	168,347	3,403	2.021	97.979
discharge_disability	168,347	0	0.000	100.000
thrombolysis_no_not_available	168,347	0	0.000	100.000
thrombolysis_no_out_of_hours	168,347	0	0.000	100.000
thrombolysis_no_scan_not_quick_enough	168,347	0	0.000	100.000
thrombolysis_no_no_reason	168,347	0	0.000	100.000
thrombolysis_no_but_haemorrhagic	168,347	0	0.000	100.000
thrombolysis_no_but_time_window	168,347	0	0.000	100.000
thrombolysis_no_but_comorbidity	168,347	0	0.000	100.000
thrombolysis_no_but_medication	168,347	0	0.000	100.000
thrombolysis_no_but_refusal	168,347	0	0.000	100.000
thrombolysis_no_but_age	168,347	0	0.000	100.000
thrombolysis_no_but_improving	168,347	0	0.000	100.000
thrombolysis_no_but_too_mild_severe	168,347	0	0.000	100.000
thrombolysis_no_but_time_unknown_wake_up	168,347	0	0.000	100.000
thrombolysis_no_but_other_medical	168,347	0	0.000	100.000
onset_to_thrombolysis_time	168,347	0	0.000	100.000
stroke_team_id	168,347	0	0.000	100.000

Table S3: Data Summary: Missing Values by Variable

Variable	Total Patients	Missing Values	Missing (%)	Complete (%)
id	168,347	0	0.000	100.000
stroke_team	168,347	0	0.000	100.000
age	168,347	0	0.000	100.000
male	168,347	0	0.000	100.000
onset_to_arrival_time	168,347	0	0.000	100.000

Continued on next page

Table S3 – *continued from previous page*

**Variable**	**Total Patients**	**Missing Values**	**Missing (%)**	**Complete (%)**
precise_onset_known	168,347	0	0.000	100.000
onset_during_sleep	168,347	0	0.000	100.000
month	168,347	0	0.000	100.000
year	168,347	0	0.000	100.000
weekday	168,347	0	0.000	100.000
arrival_time_3_hour_period	168,347	0	0.000	100.000
arrival_to_scan_time	168,347	0	0.000	100.000
scan_to_thrombolysis_time	168,347	0	0.000	100.000
congestive_heart_failure	168,347	0	0.000	100.000
hypertension	168,347	0	0.000	100.000
atrial_fibrillation	168,347	0	0.000	100.000
diabetes	168,347	0	0.000	100.000
prior_stroke_tia	168,347	0	0.000	100.000
afib_antiplatelet	168,347	0	0.000	100.000
afib_anticoagulant	168,347	0	0.000	100.000
new_afib_diagnosis	168,347	0	0.000	100.000
any_afib_diagnosis	168,347	0	0.000	100.000
prior_disability	168,347	0	0.000	100.000
stroke_severity	168,347	0	0.000	100.000
nihss_complete	168,347	0	0.000	100.000
nihss_arrival_loc	168,347	0	0.000	100.000
nihss_arrival_loc_questions	168,347	2,802	1.664	98.336
nihss_arrival_loc_commands	168,347	2,610	1.550	98.450
nihss_arrival_best_gaze	168,347	3,258	1.935	98.065
nihss_arrival_visual	168,347	4,264	2.533	97.467
nihss_arrival_facial_palsy	168,347	2,645	1.571	98.429
nihss_arrival_motor_arm_left	168,347	2,612	1.552	98.448
nihss_arrival_motor_arm_right	168,347	2,615	1.553	98.447
nihss_arrival_motor_leg_left	168,347	2,748	1.632	98.368
nihss_arrival_motor_leg_right	168,347	2,769	1.645	98.355
nihss_arrival_limb_ataxia	168,347	4,723	2.806	97.194
nihss_arrival_sensory	168,347	4,119	2.447	97.553
nihss_arrival_best_language	168,347	2,775	1.648	98.352
nihss_arrival_dysarthria	168,347	3,295	1.957	98.043
nihss_arrival_extinction_inattention	168,347	3,403	2.021	97.979
discharge_disability	168,347	0	0.000	100.000
thrombolysis_no_not_available	168,347	0	0.000	100.000
thrombolysis_no_out_of_hours	168,347	0	0.000	100.000
thrombolysis_no_scan_not_quick_enough	168,347	0	0.000	100.000
thrombolysis_no_no_reason	168,347	0	0.000	100.000
thrombolysis_no_but_haemorrhagic	168,347	0	0.000	100.000
thrombolysis_no_but_time_window	168,347	0	0.000	100.000
thrombolysis_no_but_comorbidity	168,347	0	0.000	100.000
thrombolysis_no_but_medication	168,347	0	0.000	100.000
thrombolysis_no_but_refusal	168,347	0	0.000	100.000

Continued on next page

Table S3 – *continued from previous page*

Variable	Total Patients	Missing Values	Missing (%)	Complete (%)
thrombolysis_no_but_age	168,347	0	0.000	100.000
thrombolysis_no_but_improving	168,347	0	0.000	100.000
thrombolysis_no_but_too_mild_severe	168,347	0	0.000	100.000
thrombolysis_no_but_time_unknown_wake_up	168,347	0	0.000	100.000
thrombolysis_no_but_other_medical	168,347	0	0.000	100.000
onset_to_thrombolysis_time	168,347	0	0.000	100.000
stroke_team_id	168,347	0	0.000	100.000

S1.2 Data cleaning

S1.2.1 Ambulance times

Ambulance times a renot used in the machine learning model.

Of 360,381 records, 108,499 had some ambulance times data. Where data was incomplete or suspected to be in error, all ambulance times were set to 'not recorded'. This applied to:

- 15,529 records had incomplete ambulance time data
- 6,609 records had negative time periods
- 285 records had ambulance times, other wait time at hospital, of zero
- 9 records had ambulance times, but were not recorded in SSNAP as arriving by ambulance
- 12 records had a time from call to ambulance arrival of more than 24 hours
- 3 records had an on-scene time of more than 12 hours
- 21 records had a travel time to hospital of more than 6 hours
- 61 records had a wait time at hospital of more than 12 hours

After cleaning, 85,701 records had complete and *reasonable* ambulance data.

S1.3 Hospital data cleaning

The following records were modified:

- 61 records had negative onset-to-arrival times; there were set to unknown
- 1 record had inconsistent anti-coagulant data (anti-coagulation was recorded as 'no' but a particular subtype of anticoagulant was recorded). This was set to anticoagulation 'not recorded'.
- 10 records had inconsistent death data (where death is recorded in multiple fields) - in this case death in all fields was set as 'not recorded'.
- 1388 records had missing stroke type; these records were removed.

S1.4 Cleaned data

Of 360,381 records, 358,993 remained after cleaning. 23,989 records (0.67% of original data) had partial data set to 'unknown' due to incompleteness, inconsistency, or suspected inaccuracies.

168,347 records were for patients who arrived at hospital by ambulance within 4 hours of known stroke onset time. These were used for the machine learning study on outcomes.

S2 Change in mRS between discharge and 6 month follow-up

Of 357,805 patients with a recorded mRS at discharge, 109,723 (30.7%) had a six month follow up. Though there is likely non-random selection of who is followed up at 6 months thrombolysis rates are similar between those with and without follow-up (11.7% *c.f.* 11.3%).

Table S4 shows that *on average*, mRS at 6 months is very close to mRS at discharge (0.12 higher), but there is variability between patients, and the average absolute change in mRS is 1.1.

Table S4: Modified Rankin Scale (mRS) chanage between discharge and 6 month follow-up

**Measure**	**Mean**	**Std**
mRS before stroke	0.867	1.252
mRS at discharge	2.061	1.427
mRS at 6 month follow-up	2.177	1.807
Change from discharge to 6 months	0.116	1.525
Absolute change from discharge to 6 months	1.095	1.067

Figure S1 shows a histogram of changes across the population. 31% had no change between discharge and 6 month follow-up, 73% had no change or a change of 1 mRS band, and 27% had a change of two or more mRS bands. S2 shows a box-plot of changes in mRS stratified by mRS at discharge. Those discharged with no disability (mRS 0) are more likely to worsen over 6 months (they cannot improve from mRS 0). Those discharged with mRS 3 are more likely to improve (have a lower mRS at 6 months), though those with higher discharge disability of mRS 4 or 5 will on average stay the same or worsen.

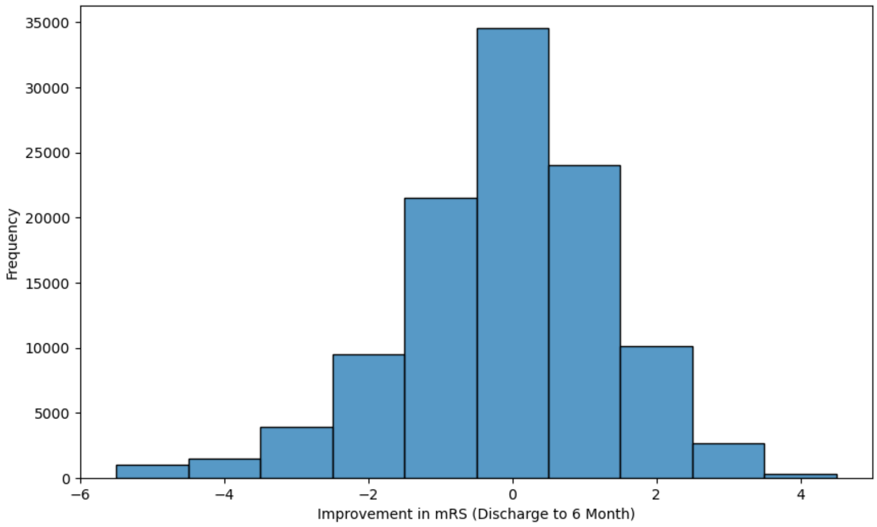

Figure S1: Improvement in mRS from Discharge to 6 Month Follow-up by Discharge mRS. A positive number indicates reducing disability.

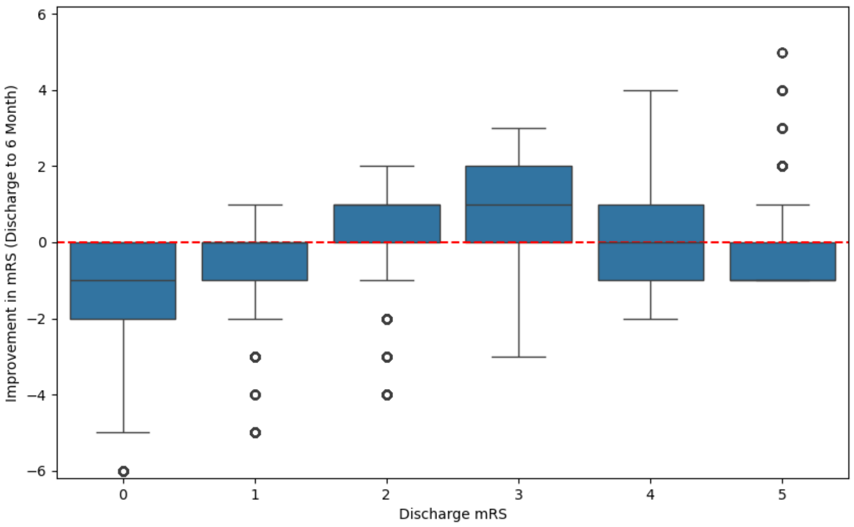

Figure S2: Improvement in mRS from Discharge to 6 Month Follow-up by Discharge mRS. A positive number indicates reducing disability.

S3 Descriptive statistics by stroke team

The following tables show statistics aggregated by stroke team.

Table S5: Descriptive statistics **for all patients** arriving at each stroke team. The table shows summary statistics across all stroke teams.

Statistic	Stroke teams	mean	Std Dev	min	25%	50%	75%	max
Yearly admissions	119	509	208	95	372	489	627	1183
Age (mean)	119	74	2	65	73	75	76	78
Proportion aged 80+	119	0.40	0.06	0.20	0.36	0.40	0.44	0.51
Proportion male	119	0.53	0.02	0.47	0.51	0.53	0.55	0.60
Prior disability (mRS, mean)	119	1.02	0.25	0.29	0.87	1.03	1.21	1.60
Proportion prior disability (mRS) 0-2	119	0.81	0.05	0.67	0.78	0.81	0.84	0.97
Proportion ischaemic stroke	119	0.88	0.02	0.83	0.86	0.88	0.89	0.93
Stroke severity (NIHSS, mean)	119	7.0	1.0	4.6	6.3	7.2	7.8	9.1
Proportion with known onset	119	0.68	0.14	0.43	0.58	0.67	0.76	1.00
Onset-to-arrival time (minutes, median)	119	204	76	109	155	180	224	466
Proportion arriving within 4 hours known onset	119	0.38	0.06	0.19	0.34	0.38	0.43	0.51
Proportion with precisely known onset	119	0.33	0.11	0.01	0.28	0.34	0.39	0.63
Proportion onset during sleep	119	0.14	0.06	0.00	0.09	0.14	0.17	0.34
Proportion arrive by ambulance	119	0.78	0.07	0.47	0.76	0.79	0.82	0.92
Call-to-ambulance arrival time (minutes, median)	113	22	10	13	17	20	24	103
Ambulance on scene time (minutes, median)	113	31	3	20	28	31	33	41

Table S5: Descriptive statistics **for all patients** arriving at each stroke team. The table shows summary statistics across all stroke teams.

Ambulance conveyance time (minutes, median)	113	18	5	10	15	17	21	37
Arrival-to-scan time (minutes, median)	119	53	21	13	39	51	63	129
Proportion receiving thrombolysis	119	0.115	0.034	0.021	0.092	0.110	0.136	0.245
Scan-to-thrombolysis time (minutes, median)	119	34	10	14	28	34	41	72
Discharge disability (mRS, mean)	119	2.641	0.352	1.361	2.413	2.699	2.900	3.320
Proportion discharged mRS 0-2	119	0.524	0.095	0.293	0.454	0.522	0.594	0.799
Proportion discharged mRS 5-6	119	0.195	0.037	0.095	0.170	0.198	0.218	0.287

Table S6: Descriptive statistics for patients arriving at each stroke team, **for patients arriving within 4 hours of known stroke onset**. The table shows summary statistics across all stroke teams.

Statistic	Stroke teams	mean	Std Dev	min	25%	50%	75%	max
Yearly admissions	119	193	78	28	139	183	241	428
Age (mean)	119	75	2	66	73	75	76	79
Proportion aged 80+	119	0.41	0.06	0.23	0.37	0.41	0.45	0.57
Proportion male	119	0.53	0.03	0.45	0.51	0.53	0.55	0.64
Prior disability (mRS, mean)	119	1.04	0.25	0.37	0.88	1.04	1.22	1.60
Proportion prior disability (mRS) 0-2	119	0.80	0.06	0.66	0.77	0.81	0.83	0.95
Proportion ischaemic stroke	119	0.85	0.03	0.75	0.84	0.85	0.87	0.94
Stroke severity (NIHSS, mean)	119	8.9	1.1	6.4	8.2	9.0	9.7	11.4
Proportion with known onset	119	1.00	0.00	1.00	1.00	1.00	1.00	1.00
Onset-to-arrival time (minutes, median)	119	105	9	85	100	105	111	132
Proportion arriving within 4 hours known onset	119	1.00	0.00	1.00	1.00	1.00	1.00	1.00
Proportion with precisely known onset	119	0.62	0.17	0.02	0.54	0.66	0.75	0.91
Proportion onset during sleep	119	0.05	0.05	0.00	0.01	0.03	0.06	0.30
Proportion arrive by ambulance	119	0.89	0.07	0.54	0.87	0.91	0.93	0.98
Call-to-ambulance arrival time (minutes, median)	110	19	5	8	16	18	21	51
Ambulance on scene time (minutes, median)	110	28	4	20	26	28	31	46
Ambulance conveyance time (minutes, median)	110	17	4	9	14	16	20	28
Arrival-to-scan time (minutes, median)	119	27	11	4	21	28	34	100
Proportion receiving thrombolysis	119	0.293	0.070	0.111	0.250	0.282	0.333	0.534
Scan-to-thrombolysis time (minutes, median)	119	34	10	14	28	34	40	71
Discharge disability (mRS, mean)	119	2.803	0.353	1.507	2.609	2.837	3.039	3.663
Proportion discharged mRS 0-2	119	0.494	0.094	0.209	0.424	0.495	0.554	0.771
Proportion discharged mRS 5-6	119	0.236	0.045	0.138	0.208	0.231	0.256	0.420

Table S6: Descriptive statistics for patients arriving at each stroke team, **for patients arriving within 4 hours of known stroke onset**. The table shows summary statistics across all stroke teams.

Table S7: Descriptive statistics for patients arriving at each stroke team, **for patients arriving by ambulance within 4 hours of known stroke onset**. The table shows summary statistics across all stroke teams.

Statistic	Stroke teams	mean	Std Dev	min	25%	50%	75%	max
Yearly admissions	119	173	74	15	125	163	227	400
Age (mean)	119	75	2	66	74	76	77	81
Proportion aged 80+	119	0.43	0.06	0.24	0.39	0.43	0.47	0.62
Proportion male	119	0.52	0.03	0.45	0.51	0.52	0.54	0.60
Prior disability (mRS, mean)	119	1.10	0.25	0.46	0.94	1.09	1.26	1.66
Proportion prior disability (mRS) 0-2	119	0.79	0.06	0.65	0.75	0.79	0.83	0.93
Proportion ischaemic stroke	119	0.85	0.03	0.75	0.83	0.85	0.87	0.94
Stroke severity (NIHSS, mean)	119	9.4	1.2	6.7	8.6	9.5	10.2	12.2
Proportion with known onset	119	1.00	0.00	1.00	1.00	1.00	1.00	1.00
Onset-to-arrival time (minutes, median)	119	106	10	84	99	105	112	151
Proportion arriving within 4 hours known onset	119	1.00	0.00	1.00	1.00	1.00	1.00	1.00
Proportion with precisely known onset	119	0.62	0.17	0.02	0.54	0.65	0.75	0.92
Proportion onset during sleep	119	0.05	0.05	0.00	0.01	0.03	0.06	0.33
Proportion arrive by ambulance	119	1.00	0.00	1.00	1.00	1.00	1.00	1.00
Call-to-ambulance arrival time (minutes, median)	110	19	5	8	16	18	21	51
Ambulance on scene time (minutes, median)	110	28	4	20	26	28	31	46
Ambulance conveyance time (minutes, median)	110	17	4	9	14	16	20	28
Arrival-to-scan time (minutes, median)	119	26	11	4	20	25	33	95
Proportion receiving thrombolysis	119	0.300	0.072	0.130	0.252	0.289	0.345	0.537
Scan-to-thrombolysis time (minutes, median)	119	34	10	13	27	33	40	73
Discharge disability (mRS, mean)	119	2.926	0.352	1.867	2.717	2.928	3.150	3.819
Proportion discharged mRS 0-2	119	0.465	0.096	0.184	0.398	0.462	0.524	0.696
Proportion discharged mRS 5-6	119	0.254	0.051	0.147	0.221	0.253	0.280	0.486

S4 Feature selection

Summary of findings

The model performance with all 58 features was:

- AUC: 0.818 (std across 5 kfolds: 0.001)
- Accuracy: 0.440 (std across 5 kfolds: 0.002)
- Accuracy within one: 0.760 (std across 5 kfolds: 0.002)

We sequentially chose features up to 25 features. Each feature was chosen to maximise mean ROC-AUC over 5 k-folds. We found that once 14 features were selected, the model chose a feature (infarction) that is the same value for all patients, hence no more information was being obtained beyond this point:

1. prior_disability, AUC: 0.687
2. stroke_severity, AUC: 0.770
3. stroke_team, AUC: 0.800
4. age, AUC: 0.806
5. year, AUC: 0.811
6. nihss_arrival_loc, AUC: 0.814
7. scan_to_thrombolysis_time, AUC: 0.816
8. thrombolysis_no_but_improving, AUC: 0.817
9. nihss_arrival_best_language, AUC: 0.818
10. new_afib_diagnosis, AUC: 0.818
11. nihss_arrival_sensory, AUC: 0.819
12. atrial_fibrillation, AUC: 0.819
13. nihss_arrival_facial_palsy, AUC: 0.819
14. thrombolysis_no_but_other_medical, AUC: 0.819
15. infarction, AUC: 0.819

Features chosen for the predictive model

We included 7 key features in our model:

1. prior_disability
2. stroke_severity
3. stroke_team
4. age
5. onset_to_thrombolysis_time
6. any_afib_diagnosis
7. precise_onset_known

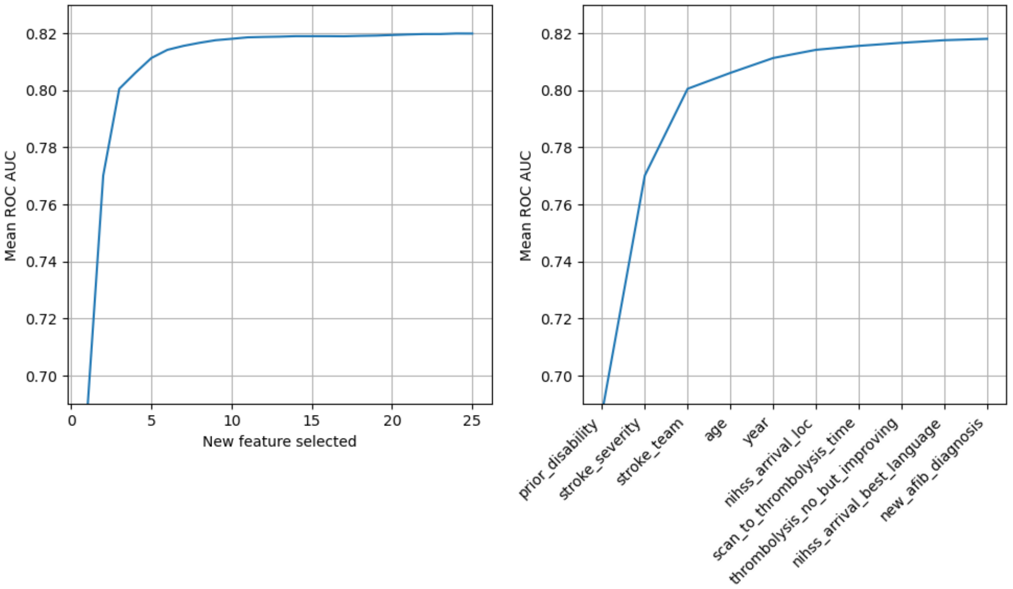

Figure S3: Improving model accuracy (Mean ROC AUC over 5 k-fold. *Left*: Accuracy with 1-25 features. *Right*: Accuracy with 1-10 features.

We did not include year as that is not a generalisable feature,

Figure S3 shows the mean ROC-AUC for the 5 k-fold model, sequentially selecting the features.

Tables S8 and S9 show model accuracy (% correct) and ROC-AUC for the models with all features and the selected 7 features.

Table S8: Accuracy (% correct) of model with all 57 features, and 7 features.

	Model accuracy (%)		Accuracy captured (%)
mRS range	All features	7 features	by 7 feature model
mRS 0	89.9	88.2	98.1
mRS 0-1	81.5	77.6	95.2
mRS 0-2	83.2	80.0	96.2
mRS 0-3	87.1	84.3	96.8
mRS 0-4	90.6	88.1	97.2
mRS 0-5	92.1	89.7	97.4

Table S9: ROC AUC of model with all 57 features, and 7 features.

	Model ROC AUC (%)		ROC AUC captured (%)
mRS range	All features	7 features	by 7 feature model
mRS 0	90.8	85.3	93.9
mRS 0-1	89.4	85.2	95.3
mRS 0-2	91.1	87.6	96.2
mRS 0-3	92.5	89.0	96.2
mRS 0-4	93.5	89.3	95.5
mRS 0-5	92.6	86.8	93.7

S5 Receiver Operating Characteristic and Sensitivity/Specificity curves

Figure S4 shows Receiver Operating Characteristic and Sensitivity/Specificity curves for each of the mRS thresholds defining a 'good' outcome.

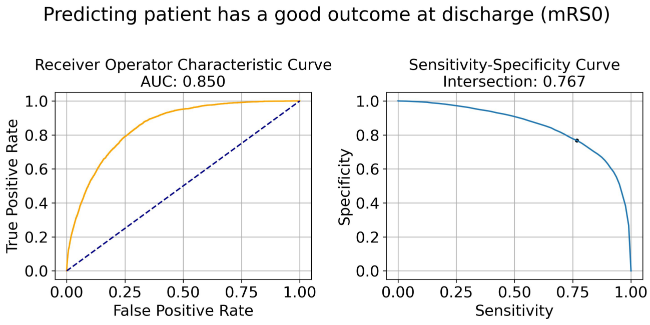

(a)

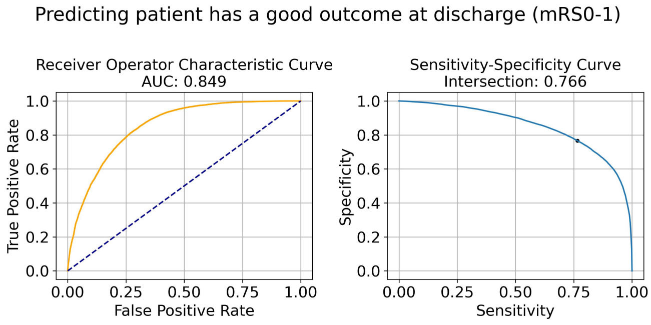

(b)

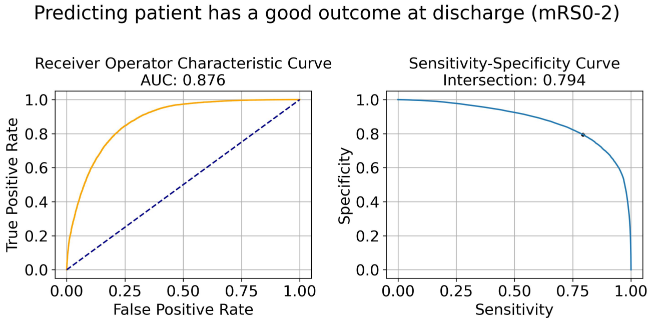

(c)

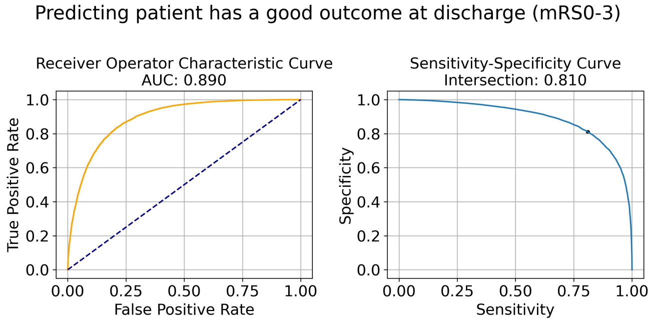

(d)

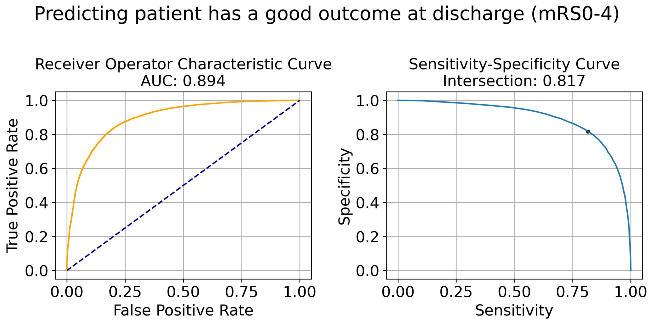

(e)

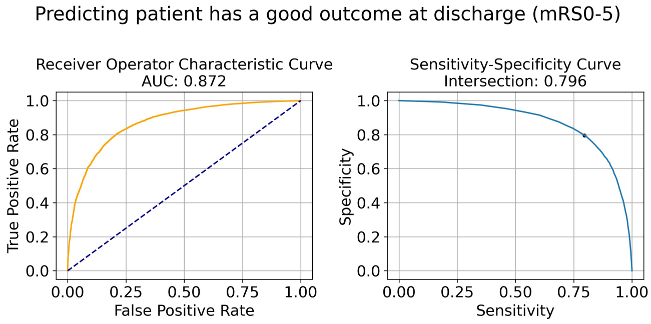

(f)

Figure S4: ROC AUC, and specificity and sensitivity plots for each of the mRS threshold levels to define a good outcome (for first kfold). Model has 7 inputs features.

S6 SHAP

Figures S5 to S9 show SHAP values for each feature across five k-fold models.

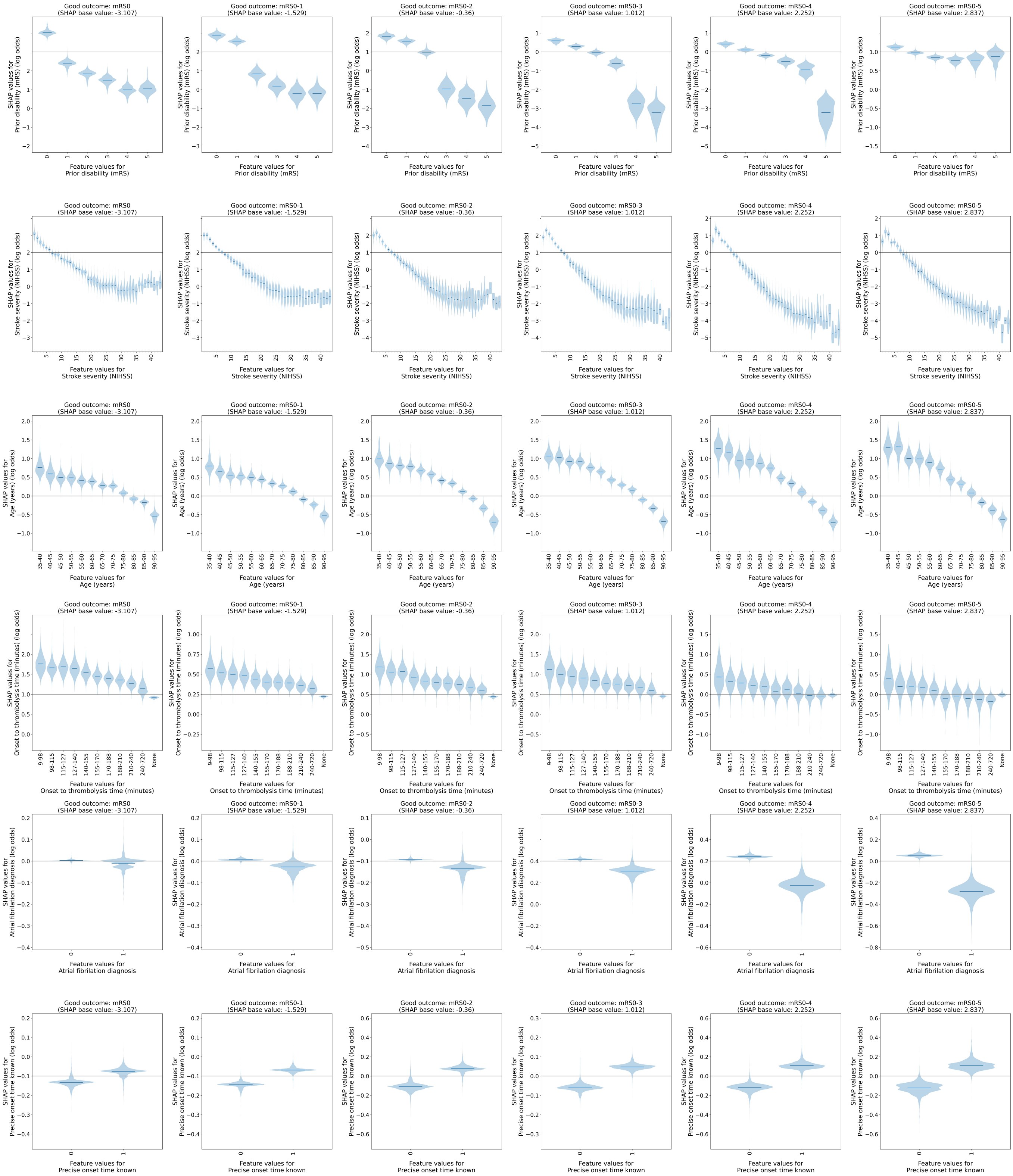

Figure S5: Violin plots show SHAP values vs feature values for each feature: Kfold 1

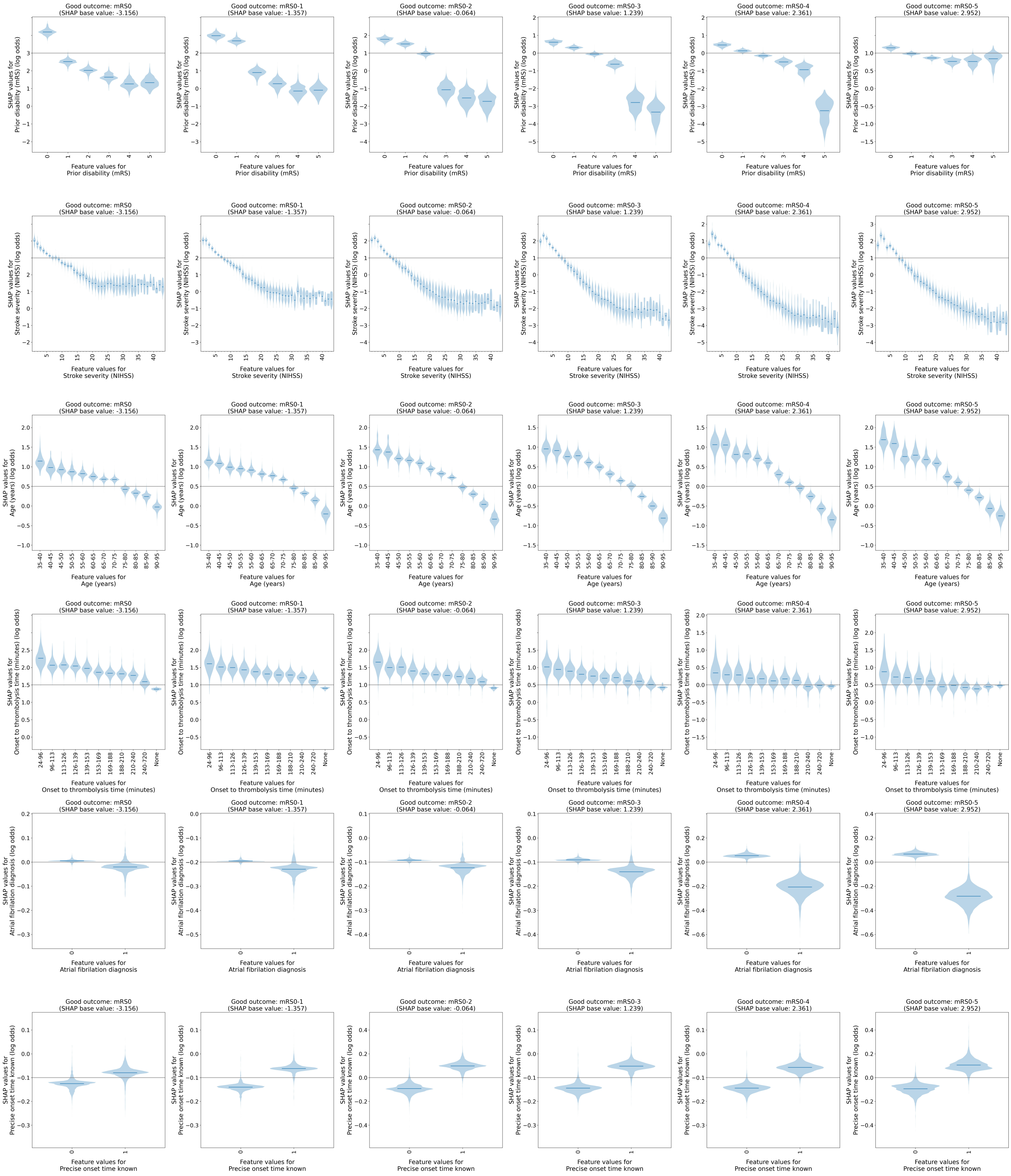

Figure S6: Violin plots show SHAP values vs feature values for each feature: Kfold 2

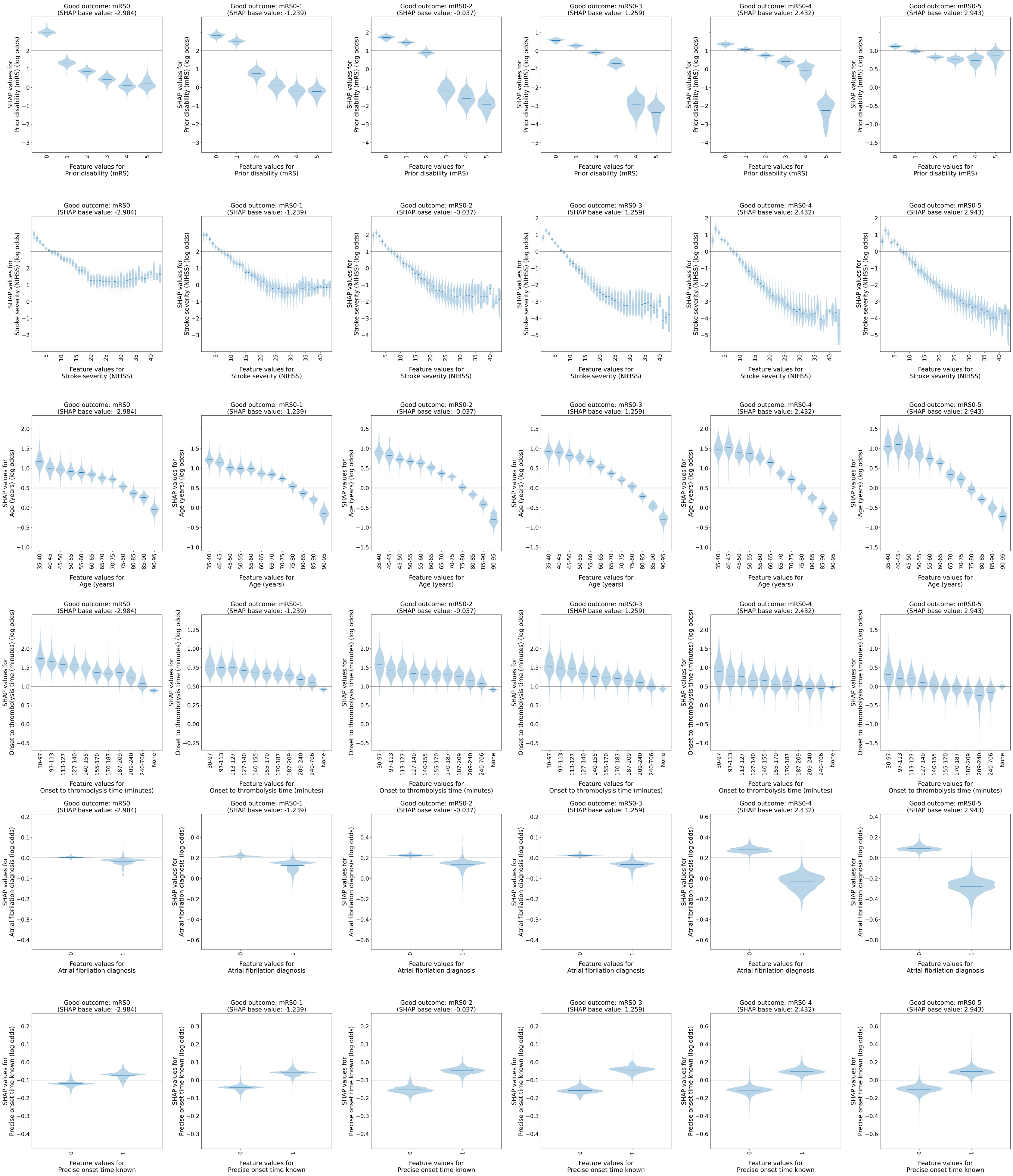

Figure S7: Violin plots show SHAP values vs feature values for each feature: Kfold 3

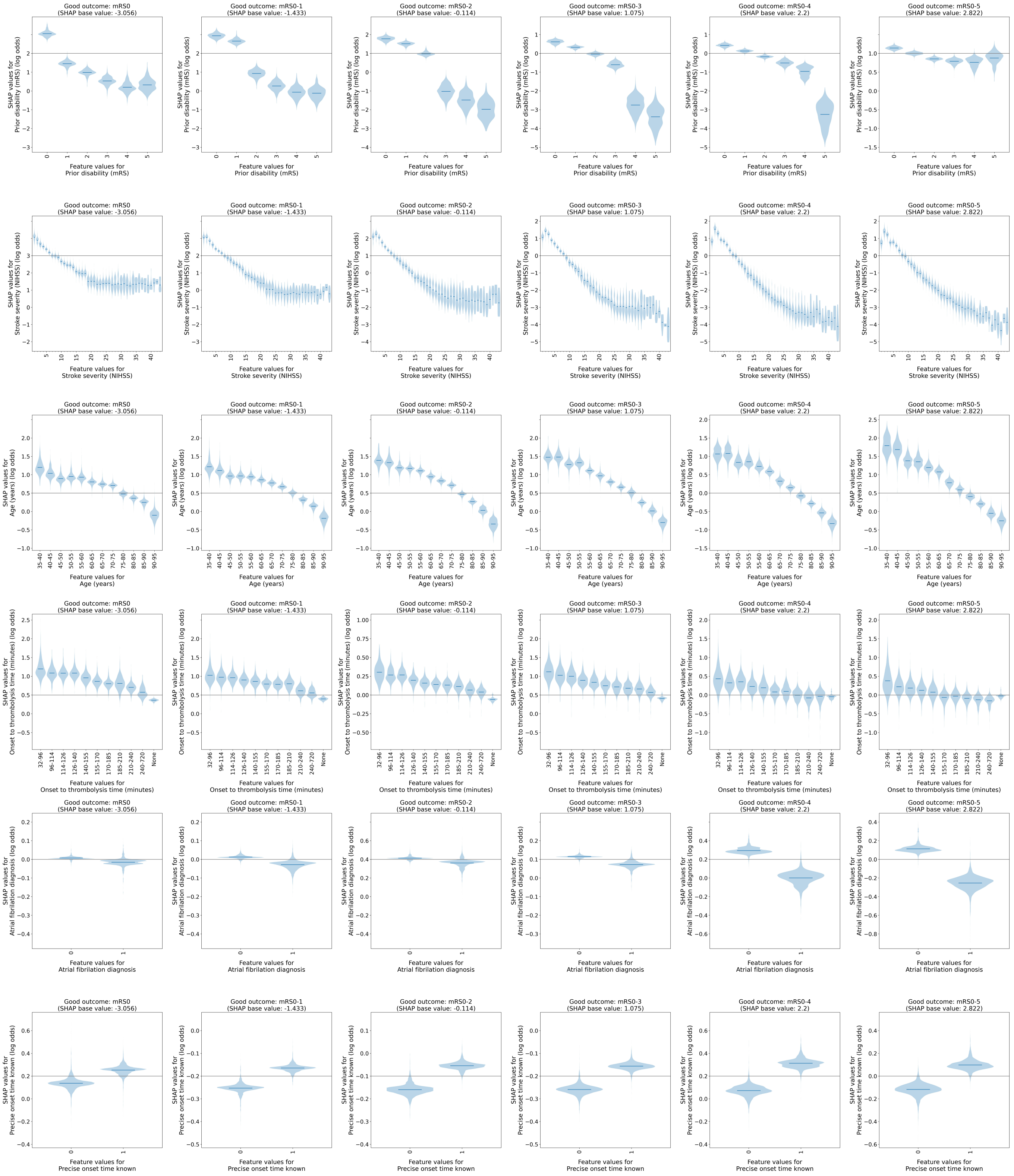

Figure S8: Violin plots show SHAP values vs feature values for each feature: Kfold 4

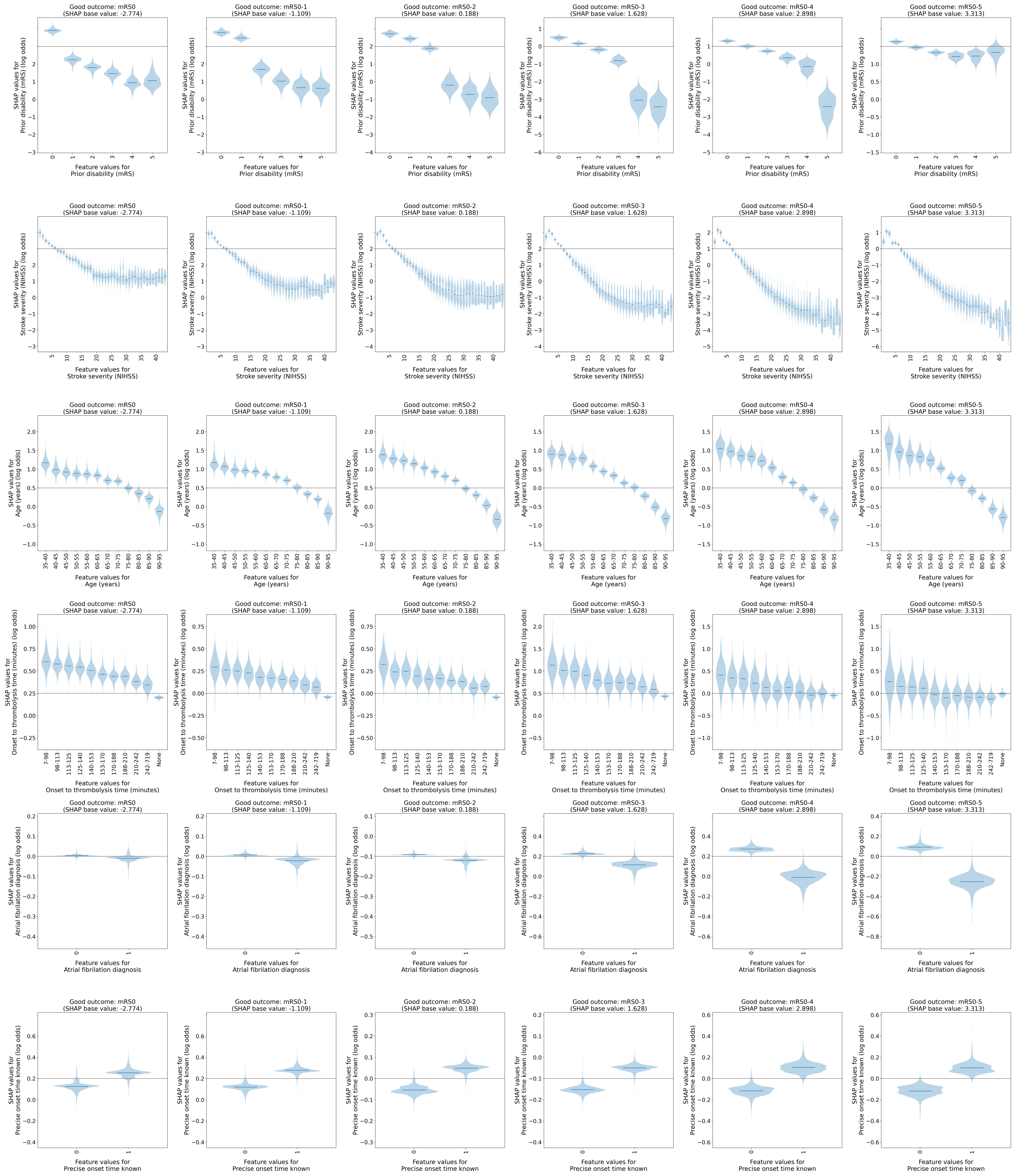

Figure S9: Violin plots show SHAP values vs feature values for each feature: Kfold 5
